# Risk factors of stroke-related sarcopenia: a systematic review and meta-analysis

**DOI:** 10.3389/fragi.2025.1452708

**Published:** 2025-01-31

**Authors:** Huan Yan, Juan Li, Lihong Xian, Yujie Li, Simin Li, Qinghua Wen

**Affiliations:** ^1^ School of Nursing, Zunyi Medical University, Zunyi, Guizhou, China; ^2^ Department of Nursing, Guizhou Provincial People’s Hospital, Guiyang, Guizhou, China; ^3^ School of Nursing, Guizhou University of Traditional Chinese Medicine, Guiyang, Guizhou, China

**Keywords:** stroke, sarcopenia, risk factors, systematic review, meta-analysis

## Abstract

**Background:**

The presence of sarcopenia at the time of stroke may deteriorate the rehabilitation and functional outcomes. There is no consensus on the factors associated with stroke-related sarcopenia because previous studies produced inconsistent and disputed results. Therefore, we screened the possible risk factors by meta-analysis.

**Methods:**

Studies published before March 2024 on risk factors with stroke-related sarcopenia were searched through PubMed, Embase, Web of Science, CINAHL, Cochrane Library, CNKI, Wan Fang, CBM, and VIP library databases. Two researchers independently screened the articles to extract the information and to evaluate their quality. Meta-analysis was then performed using Revman 5.4 software to determine the significant risk factors for patients with stroke-related sarcopenia.

**Results:**

A total of 14 studies (n = 3,113) were selected to determine the following factors that were statistically significant in patients with stroke-related sarcopenia: Age (OR = 1.04; 95% CI: 1.02, 1.06; *P* < 0.0001), tube feeding (OR = 3.98; 95% CI: 2.12, 7.47; *P* < 0.0001), pre-stroke sarcopenia (OR = 1.84; 95% CI: 1.39, 2.43; *P* < 0.0001), atrial fibrillation (OR = 1.53; 95% CI: 1.15, 2.02; *P* = 0.003), NIHSS score (OR = 1.48; 95% CI: 1.21, 1.81; *P* = 0.0001), and osteoporosis (OR = 1.801; 95% CI: 58, 2.04; *P* < 0.00001). BMI (*P* = 0.71), FOIS (*P* = 0.80), time since stroke (*P* = 0.34), and calf circumference reduction (*P* = 0.48) were not identified as risk or protective factors after stroke (*P* < 0.05).

**Conclusion:**

Our results identified various risk factors for stroke-related sarcopenia which should be considered and studied by healthcare organizations and professionals to improve the health of stroke patients.

**Systematic Review Registration:**

PROSPERO, Identifier CRD42024545757.

## 1 Introduction

The 2019 Global Burden of Disease (GBD) (GBD, 2019 Stroke Collaborators, 2021) declared stroke as the globe’s leading cause of death, and it ranks third when combined with disability. The number of strokes in rehabilitation departments usually ranges from 21% to 69% (Liu et al., 2020). Some of the affected patients experience reduced muscle mass on the paralyzed or healthy side, or even throughout the body with a concomitant decrease in muscle strength, presenting with stroke-related sarcopenia (SRS) (Li et al., 2020). With the progressively increasing aging process, the prevalence of sarcopenia is also increasing (Mas et al., 2020), ranging from 14% to 33% with a high prevalence of SRS (14%–54%).

Reportedly, the incidence of SRS not only increases the risk of further complications such as cognitive impairment, falls, functional decline, diabetes, and depression (Yuan and Larsson, 2023; Rodrigues et al., 2022; Izzo et al., 2021; Li Z. et al., 2022; Lisco et al., 2023) but also leads to the development of various metabolic disorders and increases the likelihood of cardiovascular disease (CVD) (Damluji et al., 2023). In addition, patients with SRS suffer from severe neurological damage, poor nutritional status, and lack of self-care ability (Nakanishi et al., 2021), which seriously affects their quality of life, thereby increasing the medical costs, which substantially impacts the medical burden of the family and the public health expenditure (Cui et al., 2023). Therefore, identifying the possible risk factors of SRS is essential for prognosis to reduce its associated adverse effects.

The established causes of sarcopenia are multifaceted and include ageing (Lisco et al., 2023), socio-demographic factors (Simsek et al., 2019), lifestyle (Bruyère et al., 2022), and multiple health conditions (Beaudart et al., 2014). However, previous studies have presented distinct findings on the factors associated with sarcopenia, sometimes with inconsistent and controversial results. Currently, no consensus on the factors associated with sarcopenia exists. Prevention of sarcopenia will be of special significance not only for improving the health of stroke patients but also for promoting public health.

Therefore, we systematically reviewed the factors associated with SRS through a meta-analysis to identify the significant factors and to support futuristic appropriate interventions to reduce sarcopenia and its negative effects, thereby improving the quality of life and health of stroke patients.

## 2 Materials and methods

This study followed the guidelines of the Preferred Reporting Items for Systematic Reviews and Meta-Analyzes (PRISMA) (Page et al., 2021) and the Meta-Analysis of Observational Studies in Epidemiology (Stroup et al., 2000). Additionally, it has been registered with PROSPERO (No. CRD42024545757).

### 2.1 Search strategy

Two researchers independently searched the following databases from inception to March 2024: PubMed, Embase, Web of Science, CINAHL, Cochrane Library, CNKI database, Wan Fang database, CBM databases, and VIP database. The search terms included stroke, cerebrovascular stroke, cerebrovascular disease, cerebrovascular accident, cerebral apoplexy, cerebral stroke, ischemic stroke, brain stroke, brain infarction, cerebral infarction, brain apoplexy, wind stroke, CVA, CVAs, cerebrovascular apoplexy, apoplexy, infarction, cerebrovascular stroke, brain vascular accident, acute stroke, cerebral arterial thrombosis, cerebral ischemic stroke, cerebral hemorrhage, intracerebral hemorrhage, hemorrhagic stroke, sarcopenia, sarcopeni*, muscle loss, muscle wast*, and muscle atroph*. To discover further research opportunities, we also screened the reference lists of the included articles. If necessary, we contact the authors to acquire supplementary information.

### 2.2 Inclusion and exclusion criteria

The following inclusion criteria were considered: (GBD, 2019 Stroke Collaborators, 2021) Observational study of alterations in stroke combined with sarcopenia (Liu et al., 2020); A clear description of the type of study and experimental methodology (Li et al., 2020); Accompanied by odds ratio (OR; 95% Confidence Interval or CI) data or could be transformed into OR (95% CI) data.

Articles with the following criteria were excluded: (GBD, 2019 Stroke Collaborators, 2021): Review articles, reviews, case reports, expert consensus, or guidelines (Liu et al., 2020); Agency for Healthcare Research and Quality (AHRQ) scores <4 (Hu et al., 2021) or the Newcastle-Ottawa Scale (NOS)scores <6 (Fang et al., 2023); (Li et al., 2020) Articles with incomplete or unusable data (Mas et al., 2020); Studies where the diagnostic criteria for sarcopenia were not clearly reported.

### 2.3 Selection of articles

Initially, two researchers independently screened the title and abstract of articles, excluding those that did not meet the requirements. Next, the full texts of the relevant articles were obtained and read for further screening. Moreover, the two researchers cross-checked the articles. A third person may intervene if there is any remaining controversy during the screening procedure.

### 2.4 Data extraction

Two researchers read the articles to extract the basic information, study characteristics, and observation indicators, which were recorded using Excel sheets. Subsequently, the extracted data was cross-checked. In case of incomplete data, the corresponding author was requested to provide the missing information; however, the article was excluded if the data was unavailable.

### 2.5 Quality assessment

Various quality assessment methods were used according to the nature of the studies. The 9-point NOS scale was used to evaluate prospective cohort and case-control studies, and a score of >6 was regarded as a high-quality study. The AHRQ was used to evaluate cross-sectional studies, and those with scores of <4, 4–7, and above 7 were considered to be of low, moderate, and high qualities, respectively.

### 2.6 Statistical analysis

Extracted data were subjected to meta-analysis using Revman 5.4, and count data were expressed as OR and their 95% CI. The heterogeneity of the included articles was estimated using the *I* ^*2*^ and Cochran’s Q statistics. Heterogeneity was significant when *I* ^*2*^ ≥ 50 and analyzed using a random-effects model while a fixed-effects model was used when *I* ^*2*^ < 50% or heterogeneity was non-significant. The publication bias was assessed using visual funnel plots. Specifically, the asymmetrically distributed funnel plots indicated no publication bias, and vice-versa (Sterne and Egger, 2001). Sensitivity analyses were performed by sequentially eliminating the individual studies to determine the reliability and stability of the findings.

## 3 Results

### 3.1 Search results

**FIGURE 1:**
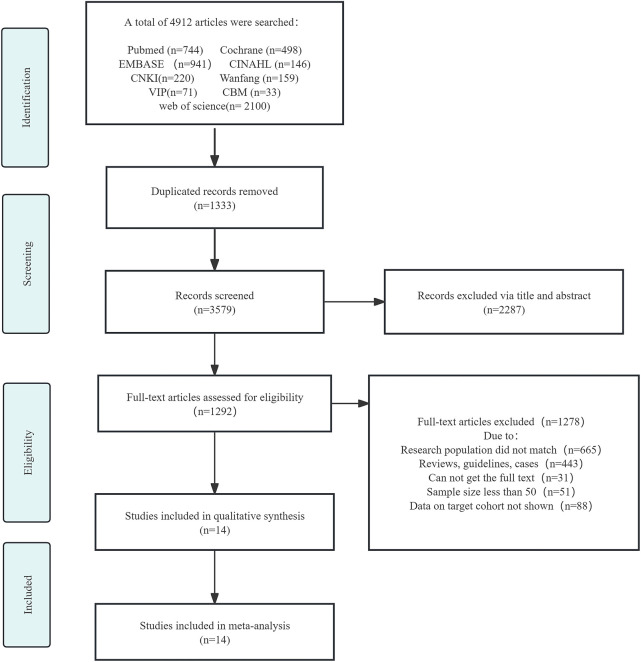
Flow chart depicting the screening and selection of the articles.

A total of 4,912 articles were retrieved of which 3,579 were selected after removing duplicates. Further, based on the titles and abstracts, we selected 1,292 studies for full-text assessment. Ultimately, 1,278 articles were excluded and 14 were included. The flowchart of literature screening is shown in Figure 1.

### 3.2 Basic characteristics of included articles

The meta-analysis included three Chinese and 11 English articles. The final selection comprised six prospective cohort studies, four cross-sectional studies, and four case-control studies, with a total sample size of 3,113 cases. The NOS scores of prospective cohort and case-control studies ranged from 7 to 9, indicating a high quality. The AHRQ scores of four cross-sectional studies ranged from 4 to 6, indicating a moderate quality. The overall score indicated that the article quality included in this study was relatively high. (Table 1). In addition, we summarized the diagnostic parameters and corresponding cut-off values for the included articles in Table 2.

**TABLE 1:** Summary of basic characteristics and risk factors of included articles.

Authors	Country	Year of publication	Type of research	Age	Gender (Male/Female)	BMI	Sample size	Risk factors assessed	NOS/AHRQ scores
Chen et al. (2023)	China	2023	Prospective cohort study	66.27 ± 3.26^a^	46/34	23.52 ± 2.17^a^	80	①②⑦	7
Wang et al. (2020)	China	2020	Case-control study	71.41 ± 5.74^a^	75/65	—	140	⑦	7
Yan-li et al. (2023)	China	2023	Prospective cohort study	65.2 ± 6.1^a^	108/92	21.46 ± 1.67^a^	200	③⑨⑩	8
Li et al. (2022b)	United States	2022	Cross-sectional study	57.4 (11.9)^b^	16/12	—	28	⑥	4
Tanaka et al. (2024)	Japan	2024	Case-control study	70.8 ± 12.4^a^	35/16	23.2 ± 3.6^a^	51	⑤	7
Nimphan et al. (2022)	Thailand	2022	Cross-sectional study	60 (9)^b^	25/12	—	37	②⑧	6
Kim and Choi (2023)	Korea	2023	Prospective cohort study	76.6 ± 6.6^a^	58/94	23.9 (21.4–26.8)^c^	152	①③④⑧	8
Yao et al. (2022)	China	2022	Case-control study	57 (18–88)^c^	179/80	—	259	①④	8
Nozoe et al. (2021)	Japan	2021	Case-control study	76 (11)^b^	187/137	22.5 (4.2)^b^	324	③⑨	7
Aydin et al. (2021)	Turkey	2021	Cross-sectional study	64.6 ± 9.1^a^	40/41	30.8 ± 6.3^a^	81	①⑥	4
Ikeji et al. (2023)	Japan	2023	Prospective cohort study	72 (14)^b^	194/92	24 (5)^b^	286	①②③⑤	9
Imamura et al. (2023)	Japan	2023	Prospective cohort study	76 (11)^b^	167/123	23 (4)^b^	290	③⑨	8
Song et al. (2023)	China	2023	Prospective cohort study	72.0 (66.0–80.0)^c^	592/410	23.2 (4.6)^b^	1,002	①②③⑨	8
Nozoe et al. (2019)	Japan	2019	Cross-sectional study	75 (11)^b^	103/80	23 (4)^b^	183	⑨⑩	5

① Age; ② BMI; ③ NIHSS score; ④ Tube feeding; ⑤ FOIS; ⑥ Time since stroke; ⑦ Osteoporosis; ⑧ Calf circumference reduction; ⑨ Pre-stroke sarcopenia; ⑩ Atrial fibrillation; “—” means not mentioned; a: mean ± standard deviation; b: Median (Interquartile Range); c: Mean (Minimum and Maximum).

**TABLE 2:** Diagnostic parameters and cut-off values of the included articles.

Author/Year	Definition of sarcopenia	Key parameters	Cut-off values
Chen Linlin 2023	Decreased muscle mass, decreased muscle strength and/or body dysfunction	① muscle mass (CC, SMI); ② muscle strength (HGS); ③ body function (6MWT, 5STS, SPPB)	① calf circumference:<34 cm for men and <33 cm for women; SMI: ≤7.0 kg/m^2^ for men and ≤5.4 kg/m^2^ for women; ② HGS:<28 kg for men and <18 kg for women; ③ 6MWT<1.0 m/s or 5STS ≥12 s or SPPB score ≤9
Wang Jianhua 2020	Decreased muscle mass combined with decreased muscle strength	① HGS; ② SMI	① HGS: <26 kg for men and <18 kg for women; ② SMI: ≤7.0 kg/m^2^ for men and ≤5.4 kg/m^2^ for women
Li Yanli 2023	Decreased muscle mass	SMI	SMI: ≤7.0 kg/m^2^ for men and ≤5.7 kg/m^2^ for women
Li S 2022	Decreased muscle mass, decreased muscle strength and/or body dysfunction	① muscle mass (SMI); ② muscle strength (HGS); ③ body function (6MWT, TUG)	① SMI: ≤7.0 kg/m^2^ for men and ≤5.5 kg/m^2^ for women; ② HGS: <27 kg for men and <16 kg for women; ③ 6MWT < 1.0 m/s or TUG ≥ 20 s
Shota Tanaka 2024	Decreased muscle mass	SMI	SMI: ≤7.0 kg/m^2^ for men and ≤5.7 kg/m^2^ for women
C. Nimphan 2022	Decreased muscle mass, decreased muscle strength and/or body dysfunction	① muscle mass (SMI); ② muscle strength (HGS); ③ body function (6MWT, SPPB)	① SMI: ≤7.0 kg/m^2^ for men and ≤5.4 kg/m^2^ for women; ② HGS: <28 kg for men and <18 kg for women; ③ 6MWT < 1.0 m/s or SPPB score ≤9
Yeo Hyung Kim 2023	Decreased muscle mass combined with decreased muscle strength	① HGS; ② SMI	① HGS: <28 kg for men and <18 kg for women; ② SMI: ≤7.0 kg/m^2^ for men and ≤5.7 kg/m^2^ for women
Ruihong Yao 2022	Decreased muscle mass combined with decreased muscle strength	① HGS; ② SMI	① HGS: <28 kg for men and <18 kg for women; ② SMI: ≤7.0 kg/m^2^ for men and ≤5.7 kg/m^2^ for women
Masafumi Nozoe 2021	Decreased muscle strength and motor function	SARC-F	SARC-F score ≥ 4
Tu ğba Aydin 2021	Decreased muscle mass, decreased muscle strength and/or body dysfunction	① muscle mass (SMM); ② muscle strength (HGS); ③ body function (6MWT, SPPB)	① SMM: <9.2 kg/m^2^ for men and 7.4 kg/m^2^ for women; ② HGS:<27 kg for men and <16 kg for women; ③ 6MWT < 0.8 m/s or SPPB score ≤ 8
Rio Ikeji 2023	Decreased muscle mass combined with decreased muscle strength	① HGS; ② SMI	① HGS: <28 kg for men and <18 kg for women; ② SMI: ≤7.0 kg/m^2^ for men and ≤5.7 kg/m^2^ for women
Madoka Imamura 2023	Decreased muscle strength and motor function	SARC-F	SARC-F score ≥ 4
Xiaodong Song 2023	Decreased muscle strength and motor function	SARC-F	SARC-F score ≥ 4
Masafumi Nozoe 2019	Decreased muscle strength and motor function	SARC-F	SARC-F score ≥ 4

### 3.3 Meta-analysis results

#### 3.3.1 Age

**FIGURE 2:**
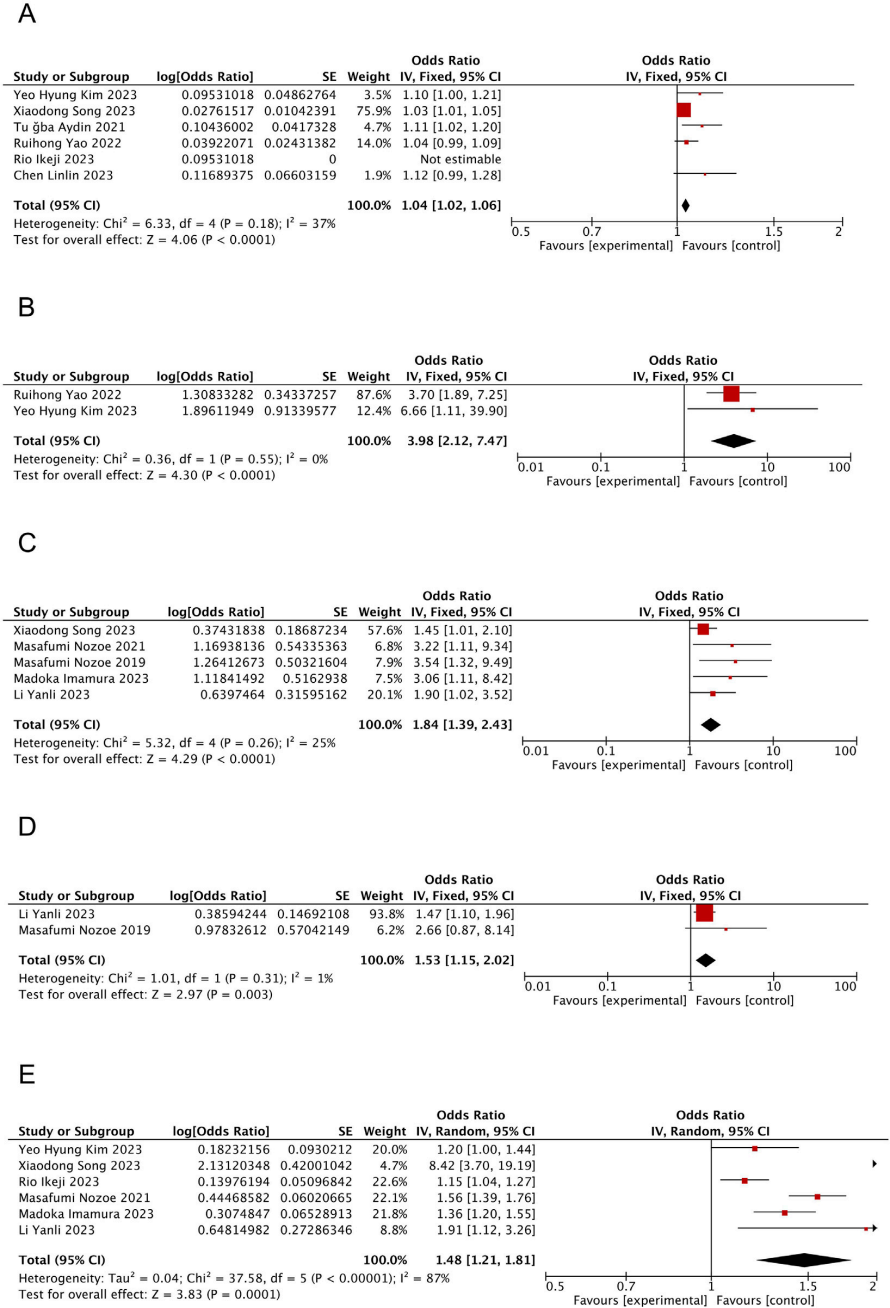
Forest plot of the meta-analysis results. **(A)** Forest plot of age factors; **(B)** Forest plot of tube feeding factors; **(C)** Forest plot of pre-stroke sacopenia factors; **(D)** Forest plot of atrial fibrillation factors; **(E)** Forest plot of NIHSS score factors.

Six studies (Chen et al., 2023; Kim and Choi, 2023; Yao et al., 2022; Aydin et al., 2021; Ikeji et al., 2023; Song et al., 2023) reported the effect of age on SRS with <50% heterogeneity between articles (*I* ^*2*^ = 37%, *P* = 0.18); however, slight heterogeneity was observed within acceptable limits. The fixed-effects model analyses showed that patients with SRS were notably older than those without SRS (OR = 1.04; 95% CI: 1.02, 1.06; *P* < 0.0001, see Figure 2A).

#### 3.3.2 Tube feeding

Two studies (Kim and Choi, 2023; Yao et al., 2022) reported the effect of tube feeding on SRS with no heterogeneity between articles (*I* ^*2*^ = 0%, *P* = 0.55). Moreover, the fixed-effects model analysis suggested tube feeding as an influential factor in patients with SRS (OR = 3.98; 95% CI: 2.12, 7.47; *P* < 0.0001, see Figure 2B).

#### 3.3.3 Pre-stroke sarcopenia

Five studies (Yan-li et al., 2023; Nozoe et al., 2021; Imamura et al., 2023; Song et al., 2023; Nozoe et al., 2019) reported the effect of pre-stroke sarcopenia on SRS with slight but acceptable heterogeneity between articles (<50%; *I* ^*2*^ = 25%, *P* = 0.26). Moreover, the fixed-effects model analyses showed that patients having sarcopenia before stroke were relatively more prone to experience SRS (OR = 1.84; 95% CI: 1.39, 2.43; *P* < 0.0001, see Figure 2C).

#### 3.3.4 Atrial fibrillation

Two studies (Yan-li et al., 2023; Nozoe et al., 2019) reported the effect of AF on SRS, with no heterogeneity between articles (*I* ^*2*^ = 1%, *P* = 0.31), and fixed-effects model analyses revealed a higher tendency of patients with comorbid AF to develop SRS (OR = 1.53; 95% CI: 1.15, 2.02; *P* = 0.003, see Figure 2D).

#### 3.3.5 NIHSS score

Six studies (Yan-li et al., 2023; Kim and Choi, 2023; Nozoe et al., 2021; Ikeji et al., 2023; Imamura et al., 2023; Song et al., 2023) reported the effect of NIHSS score on the incidence of SRS on admission, with >50% heterogeneity between articles (*I* ^*2*^ = 87%, *P* < 0.00001). The results from the random-effects model demonstrated significantly higher NIHSS scores among patients with SRS compared to those without SRS (OR = 1.48; 95% CI: 1.21, 1.81; *P* = 0.0001, see Figure 2E).

#### 3.3.6 Others

The combined effect values of the factors calculated by the software revealed that osteoporosis influenced SRS (*P* < 0.05) whereas the effect of the remaining factors remained uncertain (*P* > 0.05, see Table 3).

**TABLE 3:** Remaining factors.

Factors	Reported articles	Number of articles	Analysis mode	*I^2^* with *P* value	Overall combined OR	*P*-value
BMI	Chen et al. (2023), Nimphan et al. (2022), Ikeji et al. (2023), Song et al. (2023)	4	Random-Effects Model	89% with 0.00001	0.96 (0.76,1.20)	0.71
FOIS	Tanaka et al. (2024), Ikeji et al. (2023)	2	Random-Effects Model	72% with 0.06	0.91 (0.43, 1.91)	0.80
Calf circumference reduction	Nimphan et al. (2022), Kim and Choi (2023)	2	Random-Effects Model	88% with 0.004	2.36 (0.22, 25.70)	0.48
Time since stroke	Li et al. (2022a), Aydin et al. (2021)	2	Random-Effects Model	83% with 0.02	0.96 (0.88, 1.05)	0.34
osteoporosis	Chen et al. (2023), Wang et al. (2020)	2	fixed-effects model	41% with 0.19	1.80 (1.58, 2.04)	P < 0.001

OR, odd ratio.

### 3.4 Heterogeneity investigation and sensitivity analysis

Significant heterogeneity was observed between the six studies that reported the effect of NIHSS score; however, we sequentially excluded these studies individually to explore the source of heterogeneity but the heterogeneity persisted. This indicated the reliability and stability of the results. Moreover, different characteristics of the patients, such as the course and severity of the disease may have contributed to the heterogeneity.

### 3.5 Publication bias

**FIGURE 3:**
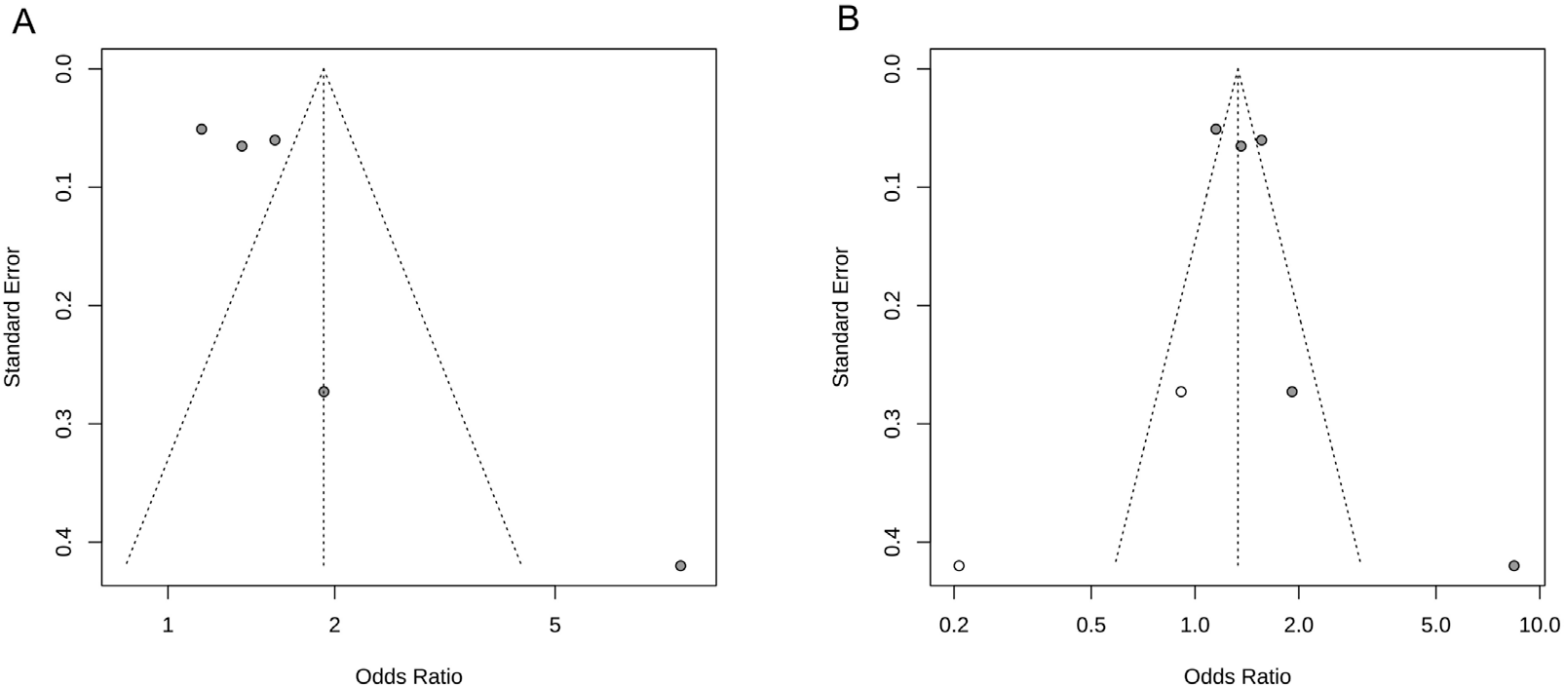
NIHSS score funnel plot. **(A)** Funnel plot of publication bias; **(B)** Adjusted funnel plot of publication bias.

The results of the study demonstrated that the heterogeneity of the NIHSS scores was >50%; therefore, we performed a funnel plot analysis of the publication bias to visually observe the signs of asymmetry in the funnel plot (see Figure 3A). Next, a trim and fill method was employed to correct the bias and to obtain the adjusted symmetrical funnel plot (see Figure 3B), thereby reporting a low risk of publication bias in the present review.

## 4 Discussion

Stroke is associated with the highest rate of disability in adults (Hacke et al., 2004). At 90 days from the onset, one-fourth of stroke survivors will experience severe motor impairment (Li et al., 2014). Although the brain is the damaged organ in stroke, the skeletal muscle is the primary cause of disability. A reduction in the number of motor units in the affected limb can be observed as early as 4 h after the stroke (Harris et al., 2001). The results showed that the risk factors associated with SRS included age, tube feeding, pre-stroke sarcopenia, AF, and NIHSS score.

According to the definition and earlier findings, sarcopenia becomes more likely as one gets older. Notably, age is an important independent predictor of strength changes (Hughes et al., 2001). A longitudinal study (Delmonico et al., 2009) investigated age-related changes in muscle mass, muscle strength, and body composition discovered that older adults experienced a progressive decline in strength and muscle mass regardless of changes in muscle mass or body weight. In addition, fat accumulation within skeletal muscles worsens with age, no matter how weight changes. Although past research has suggested that age-related changes in muscle number and mass may lead to sarcopenia, further physiological research is needed to elucidate the potential mechanisms driving the development of probable sarcopenia with age in stroke patients (Clark and Manini, 2010).

Our results suggested that tube feeding after stroke was a risk factor for the development of sarcopenia. Some stroke patients need to be fed by nasogastric tube due to dysphagia or unconsciousness at the start of their stroke. The size and location of the stroke lesion are directly related to dysphagia which may lead to swallowing-related muscle atrophy, particularly in patients with large stroke lesions. This result was consistent with a recent study that found dysphagia to be the most significant risk factor for stroke-related sarcopenia among patients undergoing rehabilitation (Yao et al., 2022). Dysphagia after stroke increases the initial stroke severity (Arnold et al., 2016). Prolonged nasogastric tube feeding may indicate severe malnutrition, further leading to loss of muscle mass and strength. Moreover, infections that impact muscle catabolism, such as aspiration pneumonia, can lead to diminished muscular strength. Thus, various factors and outcomes work together to predispose a patient to sarcopenia.

Pre-stroke sarcopenia was significantly associated with stroke prognosis. Specifically, 18% of the elderly with acute stroke had pre-stroke sarcopenia, an independent predictor of poor prognosis (Nozoe et al., 2019). A previous study showed (Ryan et al., 2017) that patients with pre-stroke sarcopenia were mostly females, had longer hospital stays and experienced higher rates of poor prognosis and prior strokes than those without sarcopenia. A possible explanation could be the decreased muscular function in patients with pre-stroke sarcopenia, making it difficult to move their arms or legs, thereby aggravating the sarcopenia symptoms and leading to severe paralysis.

Additionally, the results of our meta-analysis revealed AF was one of the significant factors. Although CVD causes muscle wasting (Proietti et al., 2022), studies on the association between AF and decompensation remain limiting (Ochi et al., 2010; Sato et al., 2020). The association between sarcopenia and AF may be due to several underlying mechanisms, particularly those associated with aging. These include changes in the cardiac conduction system, such as increased interstitial fibrosis, loss of atrial cardiomyocytes, and alterations in the distribution and function of ion channels, which may predispose individuals to AF (Pugh and Wei, 2001). In addition, AF may cause heart palpitations, fatigue and breathing difficulties, all of which can result in reduced mobility and sarcopenia (Shim et al., 2024).

Consistent with our study, a higher NIHSS score was substantially related to the risk of sarcopenia upon discharge (Kim and Choi, 2023). A prospective study reported baseline NIHSS scores as excellent predictors of post-stroke functional outcomes (Rost et al., 2016). A potential reason for this relationship could be that the severity of the stroke increases the incidence of disability, eventually leading to a reduced ability to contract on the hemiplegic and non-hemiplegic sides (García-Hermoso et al., 2018). Since patients suffering from severe stroke are more prone to develop sarcopenia, further research is necessary to elucidate the factors resulting in loss of muscle strength on the non-hemiplegic side, such as inflammation, malnutrition, and disability. Presumably, neurological factors such as insufficient nerve activation, as well as muscular factors like muscle fiber atrophy and the infiltration of fat cells leading to a decrease in overall muscle mass, work in conjunction to initiate the development of sarcopenia (Manini and Clark, 2012).

In this study, the relationship between BMI and SRS was not statistically significant, which contradicts a prior survey of potential sarcopenia among old-aged stroke survivors in the Malaysian community, where a higher BMI was strongly correlated with a lower risk of possible sarcopenia irrespective of age (Wong et al., 2022). This discrepancy may be attributed to the differences in the populations studied. Moreover, interrelationships between musculoskeletal disorders often caused by impaired gene regulation, endocrine frameworks, and close mechanical interactions (Wong et al., 2022; Kawao and Kaji, 2015; Karasik and Kiel, 2008; Laurent et al., 2016) often exist together.

## 5 Strengths and limitations

This study presented some significant findings as follows. As far as we know, this is the first systematic review and meta-analysis of risk factors for stroke-associated sarcopenia. In addition, we utilized comprehensive databases, including nine Chinese and English databases. Moreover, we employed a dual review process to improve the comprehensiveness of our findings.

Nevertheless, some limitations of the study should be acknowledged in future studies. Firstly, a high degree of heterogeneity was observed in the NIHSS scores, probably due to population, age, gender, and disease severity, or due to unavoidable heterogeneity associated with the meta-analyses of cross-sectional surveys. Secondly, the lack of standardization in the diagnostic criteria and assessment tools for SRS used in the source studies introduced measurement bias and compromised the reliability of our findings.

## 6 Conclusion

Age, tube feeding, pre-stroke sarcopenia, AF, NIHSS score, and osteoporosis are significant risk factors in patients with SRS. Moreover, BMI, FOIS, time since stroke, and calf circumference reduction are neither risk nor protective factors after stroke.

## Data Availability

The raw data supporting the conclusions of this article will be made available by the authors, without undue reservation.
